# If I Had a Hammer: The Role of Automated Impactors in Transforming Total Hip Arthroplasty Procedures and Recovery

**DOI:** 10.1016/j.artd.2025.101911

**Published:** 2026-01-12

**Authors:** Todd C. Kelley, Andrew J. Webber

**Affiliations:** The Christ Hospital, Department of Orthopaedics, Cincinnati, OH, USA

**Keywords:** THA, Impactor, Mobile sensing, Wearable technology

## Abstract

**Background:**

Impaction during total hip arthroplasty (THA) is physically demanding and places considerable stress on surgeons, contributing to fatigue and musculoskeletal injury. Automated impactor systems have emerged as a potential solution to reduce these physical demands. This study explores the dual benefits of such a system in THA, focusing on both surgeon well-being and patient recovery outcomes.

**Methods:**

The research combines wearable sensing technology and mobile sensing applications across two complementary studies. The Surgeon Exertion arm of the study assessed surgeon exertion and well-being during THA procedures performed with a traditional mallet vs an automated impactor system, measuring physiological stress markers and recovery indicators with the use of wearable sensing technology. The Postoperative Patient Outcomes arm evaluated patient recovery outcomes, leveraging mobile sensing technology to passively collect real-world data on physical activity, pain, and mood pre- and post-THA.

**Results:**

Surgeon Exertion results indicate significantly lower physiological stress markers and improved sleep quality for the surgeon when the automated impactor system was used for the day’s surgical cases compared to the traditional mallet (IMPACTOR: 108.8 bpm vs MALLET: 121.2 bpm; *P* < .0001; 7.6 h vs 6.3 h sleep; *P* < .0005). Postoperative Patient Outcomes demonstrated enhanced functional outcomes and increased daily mobility postoperatively in patients treated with the automated impactor system compared to patients treated with a traditional mallet THA (*P* = .03 for function and steps; *P* = .04 for walking distance), highlighting the device’s potential to improve patient recovery trajectories.

**Conclusions:**

The integration of an automated impactor system in THA surgeries offers significant benefits for both surgeon well-being and patient outcomes, including improved hip function, greater daily mobility, and increased walking distance. The study’s innovative use of mobile sensing and wearable technologies provides a robust framework for understanding the impacts of surgical innovations in real-world settings.

## Introduction

Orthopaedic surgery, particularly total hip arthroplasty (THA), places substantial physical and cognitive demands on surgeons. These procedures involve repetitive, forceful tasks and prolonged static postures, contributing to elevated rates of musculoskeletal injuries and burnout among orthopaedic surgeons [[1], [2], [3]]. In one survey of THA surgeons, 66% reported a work-related injury, and nearly one-third had required surgery as a result [1]. Factors such as surgeon age, years of practice, and annual case volume significantly increase risk [1].

A growing body of occupational health research shows that such high-intensity physical and cognitive workloads can impair sleep—not improve it—due to sustained sympathetic activation, elevated stress hormones, and unresolved musculoskeletal discomfort [4,5]. Studies in surgeons have demonstrated increased heart rate, cortisol levels, and reduced heart rate variability following demanding surgical shifts, all of which disrupt nighttime recovery [4]. Additionally, physical fatigue and ergonomic strain can cause sleep-disrupting discomfort, mirroring patterns seen in other high-intensity medical professions [5].

To address this occupational burden, automated surgical tools such as powered impactors have been introduced as alternatives to the traditional handheld mallet in THA. These devices are designed to reduce the physical demands of component impaction while maintaining surgical efficacy. Early studies suggest potential ergonomic and fatigue-reducing benefits associated with automated impactors [6,7], though the downstream effects on sleep and recovery remain underexplored.

While recent work has highlighted the ergonomic and health benefits of surgical technologies for operating teams, their potential to improve patient outcomes is equally important.

THA is widely accepted as an effective treatment for osteoarthritis, yet up to 23% of patients report persistent pain following the procedure [8]. This variability highlights ongoing opportunities to improve patient recovery by optimizing not only implants and techniques but also the tools used during surgery [9,10].

The present study evaluates how use of an automated impactor system influences both surgeon and patient well-being. The *Surgeon Exertion* arm of the study examines physiologic workload using wearable sensors to assess surgeon heart rate during surgery and postoperative sleep following procedures performed with either an automated impactor or a traditional mallet. The *Postoperative Patient Outcomes* arm of the study assesses recovery trajectories for patients treated with each technique, using smartphone-based sensing of mobility and mood, as well as brief, repeated self-reports of pain and function. Although previous investigations have focused primarily on ergonomic and stress-related benefits for surgeons [6,7], bench and biomechanical studies show that manual mallet impaction produces highly variable peak forces and impulse durations [[11], [12], [13], [14]]. Additionally, a recent systematic review of automated impactor systems reported more consistent force delivery and reduced surgeon fatigue without increased periprosthetic fracture risk and highlighted the need for studies linking these intraoperative advantages to postoperative patient recovery outcomes [15]. Together, these arms are designed to explore the physical and emotional effects of surgical innovation in THA.

## Materials and methods

### Participants

Participants were patients scheduled to undergo THA surgery within the Department of Orthopaedic Surgery service at the University of Cincinnati Medical Center. The service refers approximately 240 patients for conventional THA annually. All participants were recruited in accordance with the guidelines for the protection of human subjects established by the University of Cincinnati Human Research Protection Program and were paid for their participation.

Participants were eligible if they had no history of neurological injury or pathology, were community ambulatory, and had not undergone total joint arthroplasty within the previous year. Patients scheduled for staged bilateral THA procedures were eligible so long as their second surgery occurred at least 3 months after the initial procedure. All participants were required to have a compatible smartphone to enable mobile sensing app installation and data collection. This eligibility criterion was based on established smartphone ownership trends and the requirement for real-time passive and survey data collection throughout the perioperative and postoperative periods.

All THAs were performed by the same orthopaedic surgeon. Patients were randomly assigned to one of two surgery groups (IMPACTOR or MALLET) with the constraint that all surgeries performed on a given day used the same surgical instrument. This constraint was necessary to facilitate data collection for the Surgeon Exertion arm of the study. Surgeries were performed using either an automated impactor (KINCISE Surgical Automated System, DePuy Synthes, Raynham, MA) or a traditional handheld mallet. THAs were performed through a direct anterior surgical approach using press fit implants (PINNACLE acetabular component and ACTIS femoral component). For patients randomized to the impactor group, the automated impactor was used for acetabular component placement and femoral broaching portions of the procedure. For patients randomized to the mallet group, the mallet was used for acetabular component placement and femoral broaching portions of the procedure. Anesthesia was spinal anesthesia, and multimodal analgesia and rapid recovery techniques were used for all procedures. Age and body mass index did not differ statistically between groups, and no hip-related or general complications were reported in either group during the course of the study.

### Surgeon Exertion Arm

#### Design and participants

The Surgeon Exertion arm of the study included 26 THAs performed by a single orthopaedic surgeon (male, age = 49). Procedures were evenly divided between the IMPACTOR group (KINCISE Surgical Automated System) and the MALLET group (traditional handheld mallet), with only one type of surgery performed on a given day.

#### Data collection

During each surgery, the surgeon wore an Astroskin biometric shirt (Carré Technologies Inc., Montreal, QC), which continuously recorded physiologic indicators of physical stress, including heart rate, respiration rate, motion, and energy expenditure. Sleep quality was measured nightly throughout the study period using an Oura sleep ring (Oura, Oulu, Finland), which provides a total sleep score ranging from 0 to 100. Sleep scores were categorized as follows: 85-100 = optimal, 70-84 = good, 60-69 = fair, and below 60 = poor. The total sleep score is a composite index based on resting heart rate, body temperature, movement, and time spent in light, deep, and rapid eye movement (REM) sleep stages.

#### Outcomes and analyses

Analyses evaluated whether THAs performed using the automated impactor system were associated with reduced intraoperative exertion and improved postoperative sleep compared to procedures using the mallet. Physiologic and sleep data were aggregated by surgical condition (IMPACTOR vs MALLET) and compared using independent-samples *t*-tests.

Of interest was whether IMPACTOR-guided THAs reduce surgeon energy demands during surgery and whether IMPACTOR-guided THAs resulted in improved sleep patterns relative to days in which the handheld mallet was used.

### Postoperative Patient Outcomes Arm

#### Participants

The Postoperative Patient Outcomes arm of the study included 35 patients scheduled to undergo primary THA as described in the General methods section. Patients were eligible if they were scheduled for THA with the study surgeon and owned a smartphone compatible with the Mobile Activity and Emotion sensing app (MAE, Mobile Sensing Technology, Grantham, NH). All eligible patients during the study period were invited to participate. Three enrolled participants did not complete any smartphone survey prior to surgery and were therefore excluded from postoperative analyses.

#### Data collection

The Postoperative Patient Outcomes arm leveraged passive smartphone sensing and self-report surveys to assess recovery-related outcomes. Participants installed the MAE app on their smartphones, which continuously collected data via onboard sensors, including accelerometry and Global Positioning System. These data were processed using built-in computational models to infer activity states such as stationary, walking, running, driving, and cycling. In addition, the app administered brief weekly surveys assessing hip pain and function (Hip Disability and Osteoarthritis Outcome Score), mood (Patient Health Questionnaire), and pain levels.

Data collection spanned from 1 month prior to surgery through one to 3 months following the procedure. This passive approach allowed for near-continuous, low-burden tracking of real-world recovery trajectories in a home environment, complementing traditional patient-reported outcomes. The use of smartphone sensing in clinical research has been well validated in other domains [[16], [17], [18]].

#### Outcomes and analysis

Changes in patient-reported and sensed outcomes were compared between the IMPACTOR and MALLET groups using repeated-measures analysis of variance and independent-samples *t*-tests. Significance was defined as *P* < .05.

## Results

### Surgeon Exertion

**Figure 1:**
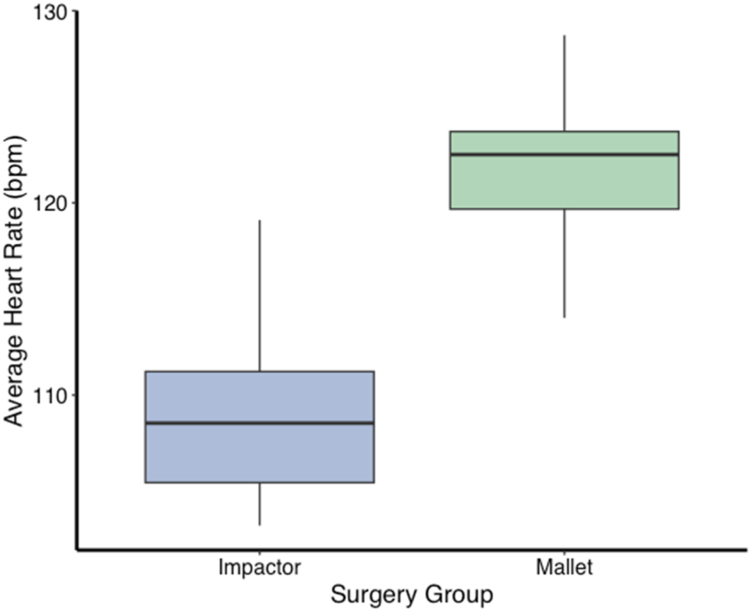
Surgeon heart rate (expressed as average beats per minute [bpm]) for Impactor (blue) and Mallet (green) surgeries. The central line within each box represents the median heart rate, while the upper and lower boundaries of the box represent the 75th and 25th percentiles, respectively, depicting the interquartile range (IQR). Whisker lines extend to the smallest and largest values within 1.5 times the IQR from the lower and upper quartiles. Differences in surgeon heart rate across the two kinds of surgery demonstrate a reduced physiological burden for impactor-assisted surgeries compared to manual, mallet surgeries.

The Surgeon Exertion arm revealed significant differences across multiple physiological metrics. Average heart rate, as measured using the Astroskin biometric shirt, was significantly higher for THAs performed with the handheld mallet (121.2 bpm) than with the automated impactor (108.8 bpm; t = −6.1; df = 24; *P* < .0001) (Fig. 1).

**Figure 2:**
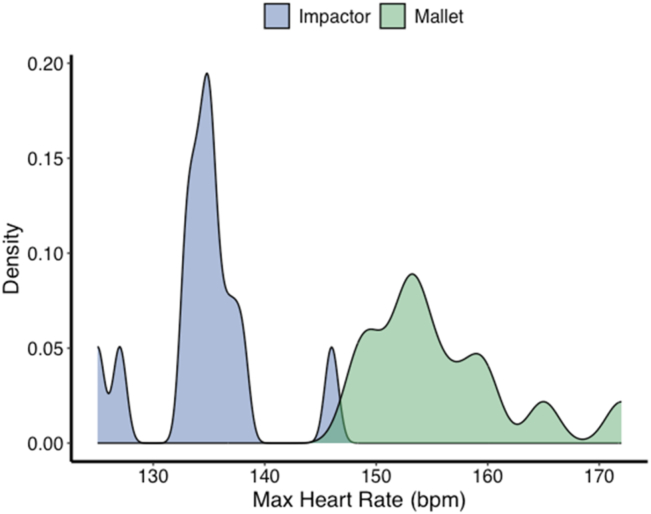
Density plots of maximum surgeon heart rates for Impactor (blue) and Mallet (green) surgeries. The two distributions illustrate the spread and central tendency of maximum heart rate values for each type of surgery. Maximum surgeon heart rate during impactor-assisted surgeries was consistently lower compared to manual, mallet surgeries, indicating a reduced physiological stress on the surgeon.

Maximum heart rate distributions were also higher for mallet-guided procedures (range: 148-172 bpm) compared to impactor-guided cases (range: 125–146 bpm; t = −9.2; df = 24; *P* < .0001) (Fig. 2).

**Figure 3:**
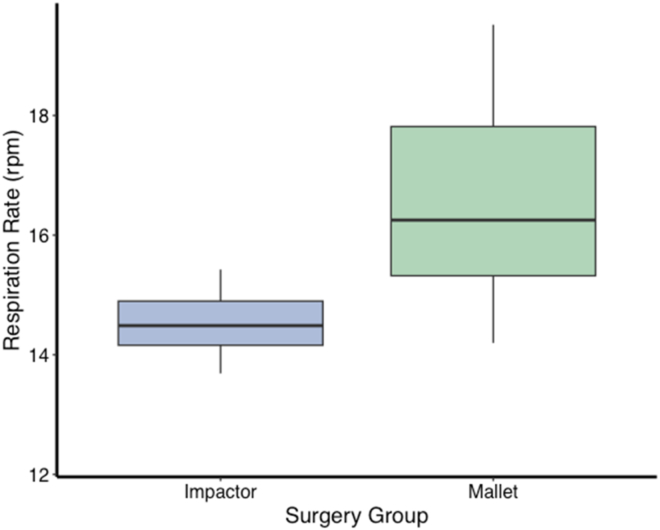
Surgeon respiration rate (expressed as average respirations per minute [rpm]) for Impactor (blue) and Mallet (green) surgeries. The central line within each box represents the median respiration rate, while the upper and lower boundaries of the box represent the 75th and 25th percentiles, respectively, depicting the interquartile range (IQR). Whisker lines extend to the smallest and largest values within 1.5 times the IQR from the lower and upper quartiles. Differences in surgeon respiration rate across the two kinds of surgery demonstrate a reduced physiological burden for impactor-assisted surgeries compared to manual, mallet surgeries.

**Figure 4:**
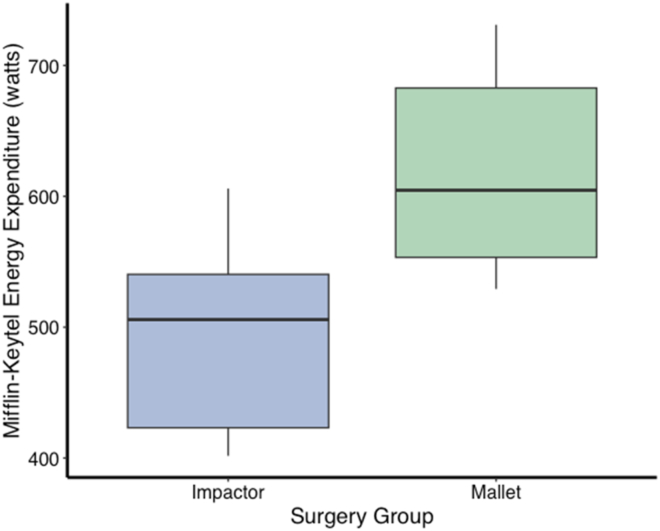
Surgeon energy expenditure (expressed as watts) for Impactor (blue) and Mallet (green) surgeries. The central line within each box represents the median energy expenditure rate, while the upper and lower boundaries of the box represent the 75th and 25th percentiles, respectively, depicting the interquartile range (IQR). Whisker lines extend to the smallest and largest values within 1.5 times the IQR from the lower and upper quartiles. Surgeon energy expenditure during the two kinds of surgery demonstrates a reduced physiological burden for impactor-assisted surgeries compared to manual, mallet surgeries.

Breathing rate and energy expenditure were significantly greater during mallet THAs compared to impactor THAs (breathing rate: mallet = 16.5 rpm, impactor = 14.3 rpm; t = −4.1; df = 24; *P* < .0005; energy expenditure: mallet = 614 W, impactor = 492 W; t = −4.4; df = 24; *P* < .0005) (Figs. 3 and 4).

**Figure 5:**
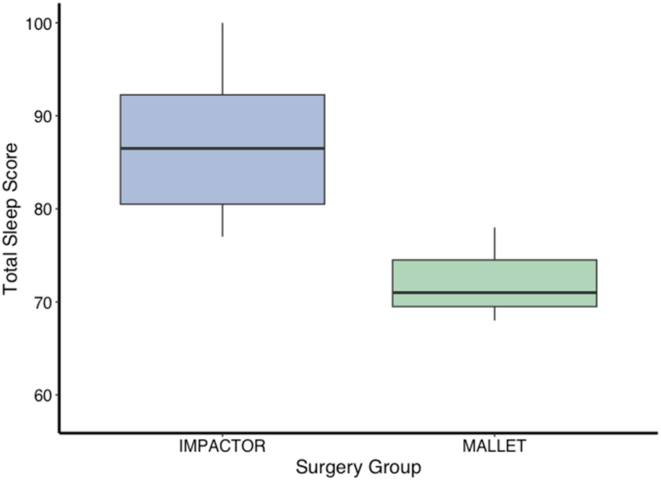
Surgeon sleep quality on evenings following Impactor (blue) and Mallet (green) surgeries. Total sleep score measures the dynamics of an individual’s resting heart rate, body temperature, movement, and time spent in specific sleep stages, including light, deep, and rapid eye movement (REM) sleep. The central line within each box represents the median total sleep score, while the upper and lower boundaries of the box represent the 75th and 25th percentiles, respectively, depicting the interquartile range (IQR). Whisker lines extend to the smallest and largest values within 1.5 times the IQR from the lower and upper quartiles. Overall sleep quality was improved following Impactor-assisted surgeries relative to manual, mallet surgeries.

Differences were also observed in sleep following surgery days. Nights following impactor-guided procedures were associated with significantly better sleep outcomes, including higher total sleep scores (impactor = 87.2, mallet = 71.0; t = 5.4; df = 19; *P* < .0001), REM sleep scores (impactor = 83.7, mallet = 67.9; t = 2.6; df = 19; *P* = .02), total sleep duration (impactor = 7.6 h, mallet = 6.3 h; t = 4.3; df = 19; *P* < .0005), and REM sleep duration (impactor = 1.5 h, mallet = 1.2 h; t = 2.7; df = 19; *P* < .01) (Fig. 5). No differences were observed in deep sleep quality or duration.

## Postoperative Patient Outcomes

Functional and behavioral measures were collected preoperatively and postoperatively in the IMPACTOR and MALLET surgery groups. Demographic and clinical characteristics for each group are summarized in Table 1. No intraoperative or immediate postoperative complications occurred, nor were any reported during routine clinical follow-ups at 2 and 6 weeks, extending to 3 months postoperatively.

**Table 1:** Patient demographics by group.

Characteristic	Impactor group (N = 17)	Mallet group (N = 18)
Age (y)	63.4 ± 6.1	62.7 ± 5.8
Body mass index	29.2 ± 4.1	29.1 ± 4.3
Sex (% female)	70.6%	66.7%

Survey compliance rates ranged from 46% to 97% across participants, with an overall average of 78%. No significant differences in compliance were observed between surgical groups or across time points.

**Figure 6:**
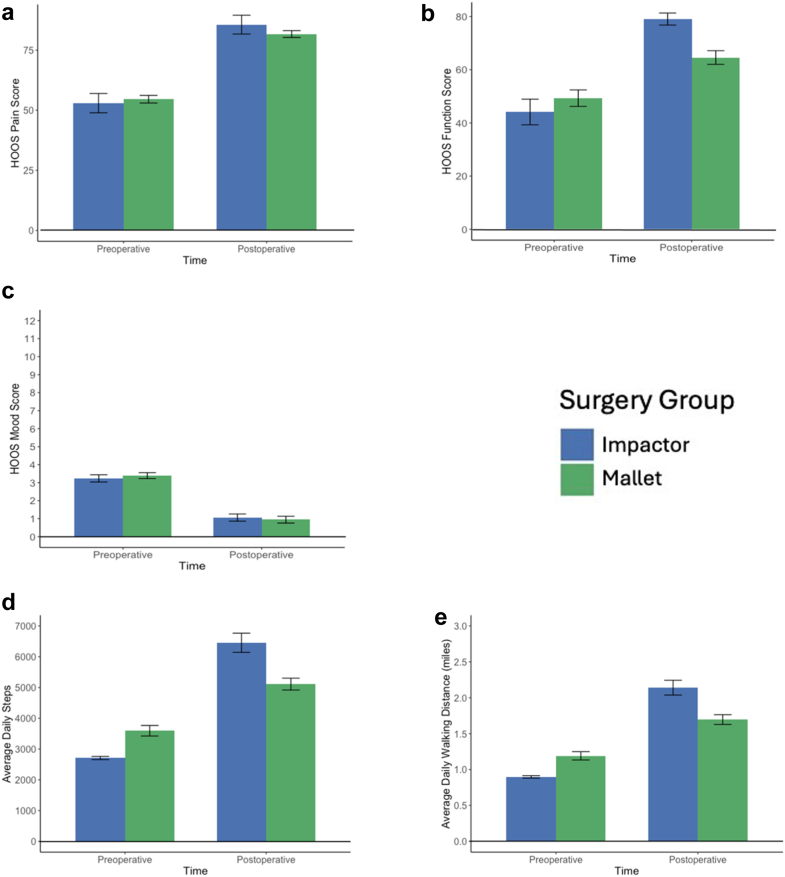
(a–c) Patient preoperative and postoperative scores on HOOS Pain (a), HOOS Function (b), and Mood (c) surveys. (d, e) Patient preoperative and postoperative mobility scores (d: daily steps; e: daily walking distance). Significant improvements were observed postoperatively across all measures following Impactor (blue) and Mallet (green) THA. The postoperative improvements in HOOS Function, daily steps, and daily walking distance were significantly greater for patients who underwent Impactor THA.

Results from repeated-measures analysis of variances revealed significant main effects of time, indicating improvement in hip pain, hip function, mood, daily steps, and walking distance following surgery (pain: F = 34.6; *P* < .001; function: F = 31.8, *P* < .001; mood: F = 27.4; *P* < .001; steps: F = 114.4; *P* < .0001; walking distance: F = 110.2; *P* < .0001) (Table 2, Fig. 6).

**Table 2:** Summary of patient outcomes by group and time.

Outcome	Group	Preoperative (mean ± SD)	Postoperative (mean ± SD)	F (time)	*P*	F (interaction)	*P*
HOOS Function	Impactor	44.1 ± 19.8	79.0 ± 9.4	31.8	<.0001	5.1	.027
	Mallet	49.3 ± 13.2	64.6 ± 10.9				
HOOS Pain	Impactor	52.9 ± 16.6	85.6 ± 16.2	34.6	<.0001	<1	n.s.
	Mallet	54.6 ± 6.7	81.7 ± 6.0				
Mood	Impactor	3.2 ± 0.8	1.1 ± 0.8	27.6	<.0001	<1	n.s.
	Mallet	3.4 ± 0.7	0.9 ± 0.8				
Daily steps	Impactor	2707 ± 214	6454 ± 1285	114.4	<.0001	4.8	.033
	Mallet	3595 ± 726	5110 ± 8112				
Walking distance	Impactor	0.9 ± 0.07	2.14 ± 0.42	110.2	<.0001	4.4	.040
	Mallet	1.19 ± 0.25	1.7 ± 0.29				

SD, Standard deviation.

There were also significant time × group interactions for hip function, daily steps, and walking distance, such that postoperative outcomes were superior in the IMPACTOR group (hip function: F = 5.1, *P* = .03; steps: F = 4.8; *P* = .03; distance: F = 4.4; *P* = .04) (Table 2, Fig. 6). Although hip pain and mood improved across the sample, these measures did not differ significantly between groups.

## Discussion

Across two complementary arms of this study, our evaluation of an automated impactor system during THA highlights its significant benefits, enhancing both surgeon well-being and patient postoperative outcomes. Findings from each arm are discussed in the context of existing literature, emphasizing broader implications for clinical practice and future research.

The Surgeon Exertion arm showed that use of the automated impactor system was associated with lower physiological stress markers, including reduced heart rate and energy expenditure, compared to traditional handheld mallet techniques. These findings align with research highlighting the substantial physical demands placed on orthopaedic surgeons during THA procedures. For instance, studies have shown that THA procedures are associated with longer operative times and increased energy expenditure compared to total knee arthroplasty [19,20]. For example, Coden et al. reported that THA required 1.2 times higher energy expenditure per patient compared to total knee arthroplasty, underscoring the significant physical demands of THA on surgeons [19].

These results are further supported by studies demonstrating that automated surgical tools can mitigate the physical toll on surgeons. Vandeputte et al. found that use of a surgical automated impactor during THA significantly improved surgeon ergonomics and reduced both physical and cognitive fatigue [6]. Similarly, Ferrari et al. reported that automated femoral broaching decreased muscle fatigue and activation in orthopaedic surgeons during simulated THA procedures [7]. Our findings add to a growing body of literature documenting the physical and psychological toll of orthopaedic practice. A recent cross-sectional study of over 1600 practicing orthopaedic surgeons reported that adult reconstruction specialists (ie, joint replacement surgeons) had among the highest rates of work-related musculoskeletal injuries, second only to general orthopaedists [21]. Notably, more than half of surveyed surgeons endorsed psychological distress since beginning practice, and nearly two-thirds reported experiencing burnout—concerning figures given their potential impact on surgeon performance, longevity, and well-being. Taken together, these findings support the potential for automated impactor systems to reduce surgeon strain, extend career longevity, and lower the risk of work-related musculoskeletal injury.

The improvement in surgeon sleep quality and duration following surgeries with the automated impactor suggests a reduced physical and cognitive toll. Sleep is essential for sustained cognitive performance and vigilance. Sleep deprivation has been shown to impair attention and executive functioning [22]. Taken together, the current findings underscore the need to further explore how surgical technologies can support surgeon health and reduce burnout risk.

The Postoperative Patient Outcomes arm demonstrated that patients undergoing THA with the automated impactor experienced greater improvements in hip function, daily steps, and walking distance during recovery. While direct comparisons to prior work are limited, these findings suggest that automated instrumentation may offer benefits for functional recovery, potentially by reducing intraoperative mechanical trauma or allowing for more consistent procedural technique. Further investigation will be necessary to determine whether these improvements are reproducible across surgical teams and settings.

Recent studies have also highlighted the potential of automated impactor systems to enhance intraoperative efficiency. For example, Thomason et al. demonstrated that use of an automated impactor during THA significantly decreased femoral broaching time by approximately 3 minutes without increasing the risk of complications [23]. These findings suggest that, beyond improving surgeon ergonomics and patient recovery trajectories, automated systems may offer additional operative efficiencies worthy of further study.

One major advantage of our approach is that it allowed us to extend data collection beyond the clinical setting and study meaningful real-world outcomes. Traditional studies often rely on in-clinic surveys and interviews, which can increase participant burden and limit data granularity. Our use of wearable and smartphone sensing technologies enabled continuous, low-burden monitoring of mood, pain, and activity and offers a novel model for orthopaedic outcomes research. This approach may allow future studies to detect long-term patterns or subtle benefits that traditional methods might miss.

### Potential limitations

While these arms offer valuable insights into the benefits of the automated impactor system in THA, several limitations warrant consideration.

First, the sample size for both surgeon and patient groups was relatively small, which may limit generalizability. This investigation was designed as a feasibility study to explore the viability of real-world physiologic and behavioral data collection using novel sensing technologies. Accordingly, all available cases were included to maximize data capture and minimize participant exclusion. Larger, multicenter studies will be important to replicate these findings across diverse clinical contexts.

Second, the study was conducted in a single setting with one surgeon. While this reduced procedural variability, it introduces potential bias related to individual technique or familiarity with the device. Future work should include multiple surgeons with varying experience levels.

The follow-up period for patient outcomes, while sufficient to capture early recovery, may not reflect longer-term trajectories. Extended follow-up studies are needed to understand the durability of the observed improvements. Other confounding factors, including call schedules, after-hours responsibilities, and activity levels outside of surgery, were not controlled and may have influenced sleep outcomes.

The reliance on wearable and smartphone sensing technologies, though innovative, may have introduced variability in data collection. While these tools enabled real-world data capture and reduced patient burden, their accuracy and reliability can vary when compared to gold standard clinical assessments. That said, app-based platforms have demonstrated significantly higher patient response rates than traditional paper methods, including in arthroplasty populations. For example, a recent study by Miller et al. found that patients using a smartphone app had significantly higher patient-reported outcome measure completion rates both preoperatively and at 1-year follow-up for hip and knee arthroplasty, with the intervention arm emerging as the strongest predictor of long-term compliance [24]. Consistent with this, we found average completion rates of 78% (range: 46% to 97%) across all postoperative outcomes, with no significant differences between groups. Although some missing data are inevitable, the use of continuous digital tracking allowed us to capture more granular behavioral and physiological data than would be feasible with conventional follow-up methods, potentially reducing recall bias and improving ecological validity.

Additionally, patients in the postoperative outcomes arm were required to own a compatible smartphone to participate. While this enabled continuous, real-world data collection via mobile sensing, it may have introduced a selection bias. Smartphone ownership is associated with higher income, education, and digital literacy and has been linked to greater healthcare engagement and follow-up adherence in other contexts [25]. Although this requirement is unlikely to have biased the group comparisons between surgical techniques reported here, it could affect the broader generalizability of patient-reported improvements following THA.

For example, in orthopaedic surgery, emerging evidence indicates that patients who engage with smartphone-based tools tend to experience improved postoperative outcomes compared to traditional care. A 2023 systematic review and meta-analysis examined 12 studies of mobile health applications in total joint arthroplasty (hip and knee replacements). It concluded that integrating smartphone apps into recovery was highly beneficial: patients using mobile apps reported significantly higher satisfaction and were more likely to adhere to rehabilitation protocols than those receiving standard care [26]. Notably, a subset of studies in the review also found that app users had fewer unplanned clinical visits—two studies documented reductions in unscheduled office or emergency department visits in the app groups. Similarly, a multicenter randomized trial in knee arthroplasty found that smartphone-based rehabilitation resulted in equivalent 1-year functional outcomes compared to standard physiotherapy, while significantly reducing in-person visits and emergency department utilization [27]. These findings suggest that smartphone-based engagement can enhance patient adherence and potentially reduce complications or concerns that lead to urgent visits. Future studies should consider strategies to reduce digital exclusion, including provisioning of devices or integrating alternative follow-up methods to ensure inclusion of lower-access populations.

## Conclusions

The present research highlights the dual benefits of an automated impactor system in THA, improving surgeon physiological outcomes and patient recovery. The Surgeon Exertion and Postoperative Patient Outcomes arms both provide early evidence that intraoperative technology can benefit the entire surgical ecosystem. As orthopaedic surgery continues to evolve, innovations that simultaneously reduce surgeon burden and enhance patient outcomes represent a promising path forward for advancing the quality of musculoskeletal care.

## Conflicts of interest

T.C. Kelley is a paid consultant for Johnson & Johnson, DePuy; and received research support from Johnson & Johnson and DePuy as a Principal Investigator; the other author declares no potential conflicts of interest.

For full disclosure statements refer to https://doi.org/10.1016/j.artd.2025.101911.

## CRediT authorship contribution statement

**Todd C. Kelley:** Writing – review & editing, Writing – original draft, Validation, Supervision, Resources, Project administration, Methodology, Investigation, Funding acquisition, Data curation, Conceptualization. **Andrew J. Webber:** Writing – review & editing, Project administration, Methodology.

## Appendix A Supplementary data

Conflict of Interest Statement for Webber

Conflict of Interest Statement for Kelley
